# Lunar synchronization of daily activity patterns in a crepuscular avian insectivore

**DOI:** 10.1002/ece3.6412

**Published:** 2020-06-09

**Authors:** Ruben Evens, Céline Kowalczyk, Gabriel Norevik, Eddy Ulenaers, Batmunkh Davaasuren, Soddelgerekh Bayargur, Tom Artois, Susanne Åkesson, Anders Hedenström, Felix Liechti, Mihai Valcu, Bart Kempenaers

**Affiliations:** ^1^ Department of Behavioural Ecology and Evolutionary Genetics Max Planck Institute for Ornithology Starnberg Germany; ^2^ Centre for Environmental Sciences, Research Group: Zoology, Biodiversity and Toxicology Hasselt University Diepenbeek Belgium; ^3^ Department of Biology Centre for Animal Movement Research Lund University Lund Sweden; ^4^ Agentschap Natuur en Bos Brussels Belgium; ^5^ Wildlife Science and Conservation Center Ulaanbaatar Mongolia; ^6^ Swiss Ornithological Institute Sempach Switzerland

**Keywords:** animal behavior, European Nightjar, foraging ecology, lunar cycle, migration, multi‐sensor logger

## Abstract

Biological rhythms of nearly all animals on earth are synchronized with natural light and are aligned to day‐and‐night transitions. Here, we test the hypothesis that the lunar cycle affects the nocturnal flight activity of European Nightjars (*Caprimulgus europaeus)*. We describe daily activity patterns of individuals from three different countries across a wide geographic area, during two discrete periods in the annual cycle. Although the sample size for two of our study sites is small, the results are clear in that on average individual flight activity was strongly correlated with both local variation in day length and with the lunar cycle. We highlight the species’ sensitivity to changes in ambient light and its flexibility to respond to such changes in different parts of the world.

## Graphical abstract

Predictable, recurring environmental changes, such as the daily light‐dark cycles, influence both nocturnal and diurnal species and have wide‐ranging effects on their behavior. We describe daily activity patterns of European Nightjars from three extremes of the species’ breeding range, during two discrete periods in the annual cycle. Our study highlights the species’ sensitivity to changes in ambient light and its flexibility to respond to such changes in different parts of the world.

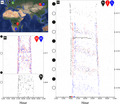

## 1 INTRODUCTION

Natural light cycles, such as day‐and‐night transitions and the lunar cycle, have been consistent over geological timescales, providing a reliable set of environmental cues that have organized ecological systems and shaped evolutionary processes (Kronfeld‐Schor et al., 2013, Gaston & Bennie, 2014; Swaddle et al., 2015). For instance, circadian and circannual rhythms of nearly all taxa are synchronized with natural light (“Zeitgeber” sensu Gwinner & Brandstätter, 2001). Variation in nocturnal light, associated with the lunar cycle influences both nocturnal and diurnal species (Miller, 2006, Kronfeld‐Schor et al., 2013, Owens & Lewis, 2018), with a wide range of effects on sleep patterns (Van Hasselt et al., 2020), reproduction (Foster, Heyward, & Gilmour, 2018; Gaston & Bennie, 2014; Jackson, 1985; York, Young, & Radford, 2014), predation risk (Griffin, Griffin, Waroquiers, & Mills, 2005; Haddock, Threlfall, Law, & Hochuli, 2019; Mougeot & Bretagnolle, 2000; Palmer, Fieberg, Swanson, Kosmala, & Packer, 2017), and foraging behavior (Kotler, Brown, Mukherjee, Berger‐Tal, & Bouskila, 2010; Ravache et al., 2020; Roeleke, Teige, Hoffmeister, Klingler, & Voigt, 2018; San‐jose et al., 2019; Da Silva, Valcu, & Kempenaers, 2015). Previous work has shown that artificial night light can disrupt these predictable lunar cues in insects (Altermatt & Ebert, 2016), amphibians (Baker & Richardson, 2006), reptiles (Brei, Pérez‐Barahona, & Strobl, 2016), mammals (Spoelstra et al., 2015), and birds (Rodríguez et al., 2017). This means that the behavioral responses of nocturnal animals might have become maladaptive, which may give cause for concern (Owens & Lewis, 2018). To assess the impact of light pollution on animal behavior, it is essential to first understand the behavioral responses of animals to periodic changes in natural light conditions (Parker & Smith, 1990; Stephens & Krebs, 1985).

**FIGURE 1:**
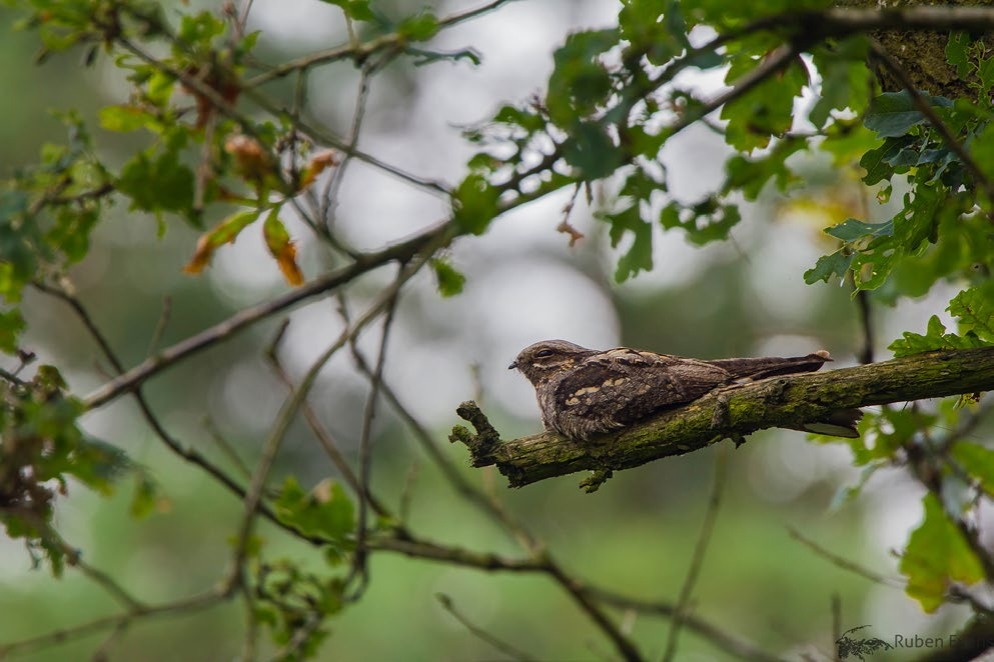
The European Nightjar (*Caprimulgus europaeus*) is a crepuscular insectivore that mainly breeds in semi‐natural, dry landscapes

**FIGURE 2:**
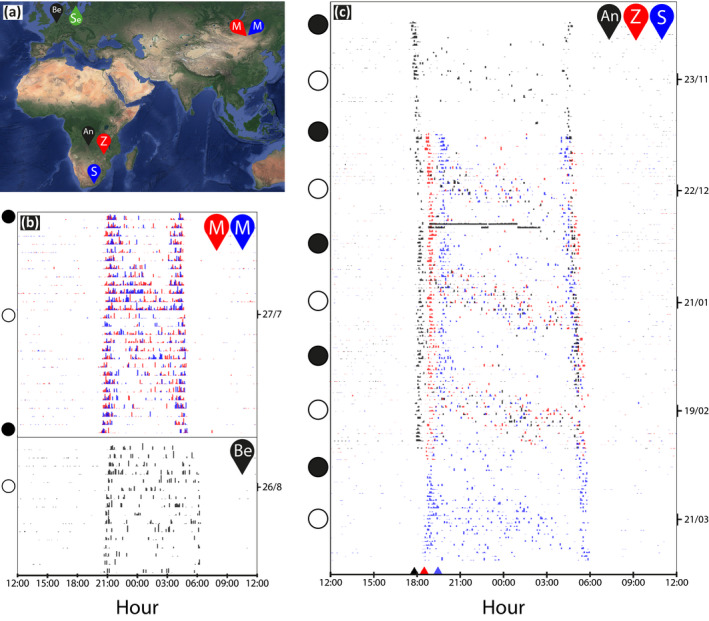
Activity patterns of three nightjars in 5‐min intervals. (a) Map of the breeding and nonbreeding areas (each color shows an individual, except green which shows the Swedish study site). Breeding sites are located in Sweden (Se), Mongolia (M), and Belgium (Be). Nonbreeding sites are located in Angola (An), Zimbabwe (Z), and South‐Africa (S). (b, c) Actograms showing daily activity (white = inactive, color = activity, height of colored bar = activity level, i.e., measured activity per 5‐min period) covering one lunar cycle during the breeding season (b; 2018) and multiple lunar cycles during the nonbreeding season (c; 2018–2019). Each horizontal bar shows one day with time on the *X*‐axis. Time is plotted in three‐hour intervals and centered around local midnight. Colored triangles show the timing of sunset (1 January) at the estimated wintering site of each individual. Open circles indicate days (nights) with full moon, closed circles show days with new moon. In C, the constant high activity at night around 31 December suggests a migratory flight

In this study, we present an analysis of the lunar‐associated behavior of European Nightjars (*Caprimulgus europaeus*; hereafter referred to as nightjar; Figure 1) by testing the hypotheses that the lunar cycle affects their nocturnal flight activity. Within the order Caprimulgiformes (Nightjars and their allies), many species are aerial insectivores adapted to a crepuscular/nocturnal lifestyle (Mayr, 2010; White, Barrowclough, Groth, & Braun, 2016). All members of the *Caprimulgidae* are visual hunters and predominantly detect flying prey against the sky (Cramp et al., 1985, Evens et al., 2018). Optimal foraging conditions for this hunting method are restricted to periods of twilight, unless nocturnal light allows prolonged foraging activities (Jetz, Steffen, & Linsenmair, 2003). Earlier observation‐based studies already suggested that several species of Caprimulgiformes synchronize their activities with the lunar cycle, showing low activity during periods of nocturnal darkness (Brigham, Gutsell, Wiacek, & Geiser, 1999) and high singing, reproductive and foraging activity around full moon (Brigham & Barclay, 1992; Holyoak, 2001; Jackson, 1985; Mills, 1986; Perrins & Crick, 1996). Despite these indications of population‐level responses to the lunar cycle, variation in activity patterns at the individual level in relation to nocturnal light conditions have not yet been quantified, except in the context of thermoregulation (e.g., Smit, Boyles, Brigham, & McKechnie, 2011) and migration (Norevik, Akesson, Andersson, Backman, & Hedenstrom, 2019). Individual nightjars became increasingly heterothermic in response to lower foraging opportunities associated with new moon periods, irrespective of relatively constant food resources (Smit et al., 2011). Similarly, better foraging opportunities during full moon periods most likely drive light‐dependent fuelling opportunities and influence the departure timing at stopover sites of migrating nightjars (Norevik et al., 2019). From the above, we expected that moonlight allows nightjars to be active longer and outside the period around dusk and dawn. As such, our study aims at testing the hypothesis that the probability that an individual is active depends on local variation in light levels. We investigated this by analyzing daily activity patterns of individuals from three distinct parts of the species’ breeding range (Mongolia, Belgium and Sweden; Figure 2a), during two discrete periods of the annual cycle (the breeding and the nonbreeding season) in relation to the lunar cycle, while controlling for variation in local day length.

## 2 METHODS

### 2.1 Field methods

We conducted fieldwork in Mongolia (48.57°N, 110.83°E), Belgium (51.06°N, 5.49°E) in 2018–2019 and Sweden (57.01°N, 15.93°E) in 2015–2017. These sites were selected because of latitudinal variation in daylength (i.e., short nights in Sweden compared to longer nights in Belgium and Mongolia) and variation in ambient light (i.e., higher nocturnal sky brightness in Belgium [20.34 mag./arcsec^2^] compared to Sweden [21.87 mag./arcsec**^2^**] and Mongolia [22 mag./arcsec**^2^**]; https://www.lightpollutionmap.info). We captured nightjars in breeding areas using ultra‐fine mist nets (Ecotone, 12 × 3m) and tape lures (Evens, Beenaerts, Witters, & Artois, 2017). We marked each individual with a unique alphanumeric ring and fitted a data logger dorsally between the wings (Evens, Conway, et al., 2017; Norevik, Akesson, & Hedenström, 2017). In total, we tagged 90 adult individuals, 20 in Mongolia, 10 in Belgium, and 60 in Sweden with a 1.2 g SOI‐GDL3pam data logger (Mongolia and Belgium (Liechti et al., 2018) or a 2.1g Multidata logger (MDL; Sweden (Norevik et al., 2019). Each logger contained sensors that recorded air pressure, ambient light intensity, and acceleration in 5‐min intervals. The SOI‐GDL3pam data loggers contained additional sensors to record air temperature in 5‐min intervals and magnetic field in 4‐hr intervals. Activity is measured as the sum of the absolute differences in acceleration on the z‐axis (SOI‐GDL3pam: a summary variable stored for each 5‐min interval (Liechti et al., 2013); MDL: a summary variable stored for each one‐hour interval (Norevik et al., 2019). In total, we recovered eleven loggers (two in Mongolia, one in Belgium and eight in Sweden; all from males; Appendix S1: Supplementary Materials T1). These low recovery rates did not allow a formal comparison of flight activity between study sites.

### 2.2 Migration data

Because nightjars use flapping flight during migration (Bruderer, Peter, Boldt, & Liechti, 2010) and remain stationary at their over‐wintering site (Evens, Conway, et al., 2017; Norevik et al., 2017), we can use the information on light intensity and acceleration to estimate nonbreeding destinations (Lisovski et al., 2019). Data from MDL loggers were preprocessed by Norevik et al. (2019). From SOI‐GDL3pam loggers, we analyzed light measurements to provide daily position estimates (Hill, 1994; Lisovski et al., 2019) using the R‐package PAMLr. Simultaneously, an automated algorithm used activity recordings to identify migratory flight bouts (minimal 60 min of high activity; for details see Liechti et al. (Liechti et al., 2018). Based on activity data and location estimates, we defined the breeding season as the period between the end of spring and the onset of autumn migration and the nonbreeding season as the period between the end of autumn migration and the onset of spring migration. We identified wintering areas for eight birds: one of the two Mongolian birds resided in South‐Africa (approximate flight distance 13,000 km) and the other in Zimbabwe (±12,000 km); the Belgian bird resided in the border region of Angola, Zambia, and the Democratic Republic of Congo (DRC; ±7,500 km); two Swedish birds wintered in Zambia (±9,500 km), another two in Botswana (±10,000 km) and one in the Democratic Republic of Congo (±8,000 km).

### 2.3 Activity data

Activity data from SOI‐GDL3pam data loggers were transformed to hourly estimates to fit the data of the MDL loggers. We subdivided daily activity data from the breeding and the nonbreeding season into three groups: daytime (from sunrise until sunset), twilight (from sunset until evening nautical twilight; from morning nautical twilight until sunrise), and night (from evening nautical twilight until morning nautical twilight). We categorized activity data into two classes: active (e.g., flight and foraging) and inactive (e.g., resting and roosting). We distinguished between these categories based on an activity threshold computed as the 97.5th percentile of daytime activity using a Linear Quantile Mixed Model (Geraci, 2014) with individual identity as random intercept, and period (winter versus. summer), time of the day and date as covariates. This threshold was chosen because nightjars remain inactive during daytime, with resting and preening as their main activities. During twilight they spent much of their time flying to forage and to commute between local sites (Evens et al., 2018; Evens, Beenaerts, et al., 2017; Mills, 1986; Wynne‐Edwards, 1930), and at night additional foraging activities can occasionally take place (Brigham et al., 1999; Evens et al., 2018).

Data on the timing of day, night, and twilight (i.e., sunset and sunrise) and the lunar cycle (i.e., altitude of the moon above the horizon and fraction of illuminated moon) were extracted for the known breeding sites and estimated nonbreeding sites and for each interval using the R‐package “suncalc.” We did not take into account variation in local weather conditions which may lead to additional noise, and hence may weaken our results rather than create systematic biases (Penteriani et al., 2013).

Of all hourly intervals, 7,434 (32%) were categorized as active, while the remaining 15,358 intervals were categorized as inactive.

We modeled nocturnal and twilight activity using Generalized Linear Mixed Models (GLMMs) with maximum likelihood using the R package glmmTMB version 0.2.3 (Brooks et al., 2017; Geraci, 2014; Team, 2019). To asses patterns of nocturnal activity, we constructed two models: 1) a model for the conditional mean containing the fraction of illuminated, visible moon (i.e., fraction of illuminated moon when above the horizon, continuous variable: percentages) and altitude of the moon above the horizon (continuous variable: radians) as the main predictors (Table 1) and 2) a zero‐inflated model which allows modeling the probability of excess zeros in the conditional part of the model. To assess patterns of crepuscular activity, we constructed a model with twilight period (categorical variable: dusk or dawn) as the main predictor (Table 1).

**TABLE 1:** Results of generalized mixed‐effect models showing effects of available moonlight or twilight on nocturnal and crepuscular activity of 11 European nightjars from Belgium, Mongolia, and Sweden at their breeding and nonbreeding sites. See Section 2 for model details

Nocturnal activity					Crepuscular activity				
*Conditional model*					*Conditional model*				
**Predictors**	**Estimate**	***SE***	***z***	***p***	**Predictors**	**Estimate**	***SE***	***z***	***p***
Intercept	−1.073	0.216	−4.97	<.0001	Intercept	1.233	0.345	3.58	<.001
Fraction visible moon^a^	1.268	0.255	4.96	<.0001	Twilight^e^	2.198	0.203	10.813	<.0001
Altitude moon^b^	1.144	0.140	8.17	<.0001	Season^c^	−0.807	0.241	−3.35	<.001
Season^c^	−0.927	0.119	−7.80	<.0001	Phase moon^f^	0.059	0.31	0.190	.849
Previous activity^d^	0.027	0.003	8.44	<.0001					
**Random effect**	**Variance**	***SD***	**Corr**		**Random effect**	**Variance**	***SD***	**Corr**	
Individual ID (random intercept)	0.289	0.537			Individual ID	0.527	0.726		
Moon within ID^g^ (random slope)	0.446	0.668	−0.66						
*Zero‐inflation model*									

Predictors	Estimate	*SE*	*z*	*p*
Intercept	−2.929	1.084	−2.70	.007

Random effect	Variance	*SD*	Corr
Individual ID	2.721	1.65	

^a^ Fraction of illuminated, visible moon.

^b^ Altitude of the moon above the horizon.

^c^ Estimates for nonbreeding compared to breeding.

^d^ Activity during the previous 60‐min period (to control for temporal autocorrelation).

^e^ Estimate for dusk compared to dawn.

^f^ Moon phase during twilight.

^g^ Fraction of illuminated, visible moon per individual.

The conditional model (nocturnal activity model) and the crepuscular activity model use a negative binomial distribution with log‐link function, whose variance was set to increase linearly with its mean. The conditional model included the following additional fixed effects: season (categorical variable: breeding or nonbreeding season) and the activity of the previous time step. The crepuscular activity model included moon phase (continuous variable: fraction of illuminated moon during twilight) and season. We included activity of the previous time step in the nocturnal activity model to control for temporal autocorrelation. We did not include time and date in our models, because they correlate with the altitude of the moon and the fraction of visible moon above the horizon. In an alternative version of the nocturnal activity model, we replaced illuminated, visible moon, and moon altitude by time (continuous variable: standardized hour per individual per night) and date (continuous variable: standardized date per season) (Appendix S1: Supplementary Materials T2). We further included individual identity as random intercept and fraction of visible moon per individual as random slope (Table 1, Appendix S1: Supplementary Material T2). The zero‐inflated model contained no predictors other than the overall mean, but contained individual identity as random intercept.

*Ethical statement*.

The Mongolian and Belgian research protocols were approved by the Mongolian (Ministry of Environment and Tourism, license numbers: 06/2564 and 06/2862) and Belgian (Agency for Nature and Forest, license numbers: ANB/BL‐FF/V18‐00086 and ANB/BL‐FF/19‐00087‐VB) authorities. The Swedish protocols follow the Swedish legislation for animal research (SJVFS 2019:9) and received the approval of the Malmö/Lund ethical committee for animal research (M33‐13). All captured individuals showed no evidence of detrimental effects of banding. The tags weighed less than 3% of the birds’ body mass, which is well below the recommended limits. Although we cannot exclude that some individuals were affected by the devices, direct observations in the field and assessment of the recaptured individuals showed no signs of negative effects. The recovery rate (11%) is lower than expected (Evens, Conway, et al., 2017; Norevik et al., 2019) and is probably caused by i) the late deployment of loggers on nonresident individuals with no fidelity to the study site in Belgium (late August 2018) and ii) a low recovery success due to bad weather conditions during a two‐week trapping session in Mongolia (July 2019).

## 3 RESULTS

**FIGURE 3:**
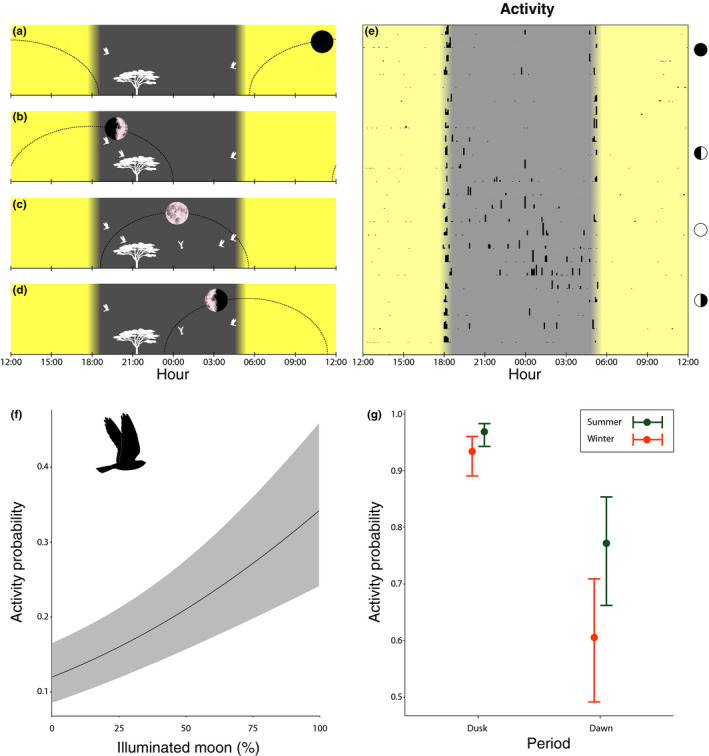
Nightjar activity in relation to light conditions. (a–e) Schematic overview of moonrise and moonset times of the Belgian bird during one lunar cycle in Angola (February 2019; full moon on the 19th; local azimuth not taken into account). (a) New moon: no moonlight available at night, (b) first quarter: moonlight available before but not after midnight, (c) full moon: moonlight available all night, and (d) last quarter: moonlight available after but not before midnight. (e) The corresponding actogram showing daily nocturnal activity (height of black bar = activity level, i.e., measured activity per 5‐min period) in relation to time. Each horizontal bar shows one day with time of day, plotted in 3‐hr intervals and centered around midnight on the *X*‐axis. Open circles indicate days with full moon, closed circles show days with new moon and half circles indicate days with first‐ or last quarter moon. (f) Relationship between the probability of nightjar activity at night in relation to the fraction of illuminated, visible moon. Shown are estimates and 95% confidence intervals based on the model in Table 1. (g) Differences in the probability of activity between dusk and dawn during the breeding season (green) and during the nonbreeding season (orange). Shown are model estimates and their 95% confidence intervals based on the model in Table 1

The nightjars’ sensitivity to changing light conditions can be seen in the actograms (Figures 2 and 3), which show the most detailed activity data (5‐min sampling intervals), collected for the Belgian and Mongolian birds. These three individuals were selected for visual purposes only. Actograms for all birds with hourly intervals are presented in the online depository.

All individuals showed clear peaks of activity around dusk and dawn, confirming the crepuscular behavior of the species (Figure 2, Appendix S1: Supplementary Materials 2). The timing of the nightjars’ crepuscular behavior matches shortening day lengths over the season in temperate zones (Figure 2b, M and S, Appendix S1: Supplementary Materials 2) and constant day length, but with latitudinal variation in the timing of dusk and dawn, in tropical zones (Figure 2a and Z, Appendix S1: Supplementary Materials 2).

**FIGURE 4:**
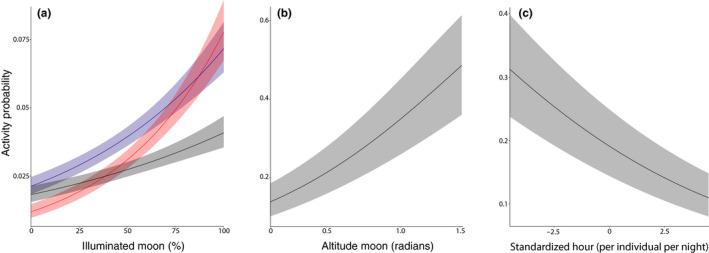
Nightjar activity in relation to light, moon altitude, and time. (a) Individual activity in relation to the fraction of visible moon. Activity data were collected at 5‐min intervals. Shown is the probability that a nightjar was active at night in relation to the fraction of illuminated moon. Each color corresponds to one individual (same color as in Figure 2: black = Belgian, blue and red = Mongolian). (b) The probability of nightjar activity at night in relation to the altitude of the moon above the horizon (expressed in radians). Shown is the estimate and 95% confidence intervals based on the model in Table 1. (c) Nightjar activity in relation to time. Time is standardized per individual per night. Shown is the probability of activity before and after midnight (estimate and 95% confidence intervals based on the model in Appendix S1: Supplementary Materials T2)

During each lunar cycle, nightjars showed a clear diagonal band of nocturnal activity with peak activities around full moon (Figure 3c, Appendix S1: Supplementary Materials 2). Nightjars were active from about the first to the last quarter of the lunar cycle (Figure 3b‐c). Formal analysis shows that the probability of nocturnal activity strongly depended on the daily (nightly) fraction of illuminated, visible moon, and the altitude of the moon above the horizon, both in the breeding and nonbreeding season (Table 1; Figure 3f). Nightjars increased their activity with increasing light (fraction of illuminated, visible moon), but the exact relationship differed between individuals (Figure 4a). Nightjars also increased their activity with increasing altitude of the moon above the horizon (Figure 4b). Furthermore, nocturnal activity was significantly higher during the breeding season and generally decreased during the night (Figure 4c).

During twilight, activity was higher at dusk than at dawn, and higher during the breeding season than during the nonbreeding season (Table 1; Figure 3g). Twilight activity was independent of the moon phase (Table 1).

## 4 DISCUSSION

Although the sample size for two of our study sites is small, the results are clear: on average, individual flight activity was strongly correlated with both local variation in day length and with the lunar cycle (Figure 2, Appendix S1: Supplementary Materials 2). This confirms our hypothesis and clearly suggests that nightjars from different populations across the breeding range are able to accurately adjust their activity (a) to seasonal changes in the timing of local dusk and dawn, in accordance with their crepuscular lifestyle, and (b) in response to local variation in available light at night. Our findings further support recent observations from a Scandinavian breeding site, where nightjars adjusted foraging flight activity at stopovers and subsequent migration activities to the lunar cycle (Norevik et al., 2019).

Nocturnal activity data display a noticeable diagonal band (Figure 2) indicating a progressive shift in the nightjars’ daily activity throughout subsequent moon phases (see also Norevik et al., 2019). This general pattern is consistent between all individuals in our study, even though they resided in different parts of the world. Flight activity of the tracked individuals corresponds to daily changes of the moon's trajectory (i.e., the moon's altitude above the horizon) and the fraction of illuminated moon (Figure 3a‐f, Appendix S1: Supplementary Materials 2). Around new moon, nightjars were largely inactive during the night (Figure 3a), whereas around full moon they seemed to fully exploit the increased ambient light by being active all night (Figure 3c). The close relationship between nocturnal activity and night light is also suggested by relatively high before‐midnight activity during a first‐quarter moon and high after‐midnight activity during a last quarter moon phase (Figure 3b and d).

The flight activity of nightjars is presumably organized by endogenous rhythms, but lunar‐associated effects are probably linked to sensing mechanisms (e.g., vision influencing nocturnal behavior) and other factors such as lunar‐associated behavior of prey and predators. Endogenous rhythms are finetuned using environmental information (Helm et al. 2017), and moonlight is an important environmental cue and *Zeitgeber* (Kronfeld‐Schor et al., 2013). Moonlight influences general activity patterns (Bachleitner, Kempinger, Wülbeck, Rieger, & Helfrich‐Förster, 2007; Youthed & Moran, 1969) and reproduction (Zantke et al., 2013), yet knowledge about endogenous circalunar rhythms in animals is limited (Kronfeld‐Schor et al., 2013, Payton & Tran, 2019). Our study confirms earlier observations of lunar‐associated behavior in nightjars (see below), but it remains to be shown whether circadian and circalunar rhythms play a role.

Variation in moonlight (affected by the moon's altitude and phase) is known to influence predation risk, foraging behavior, habitat use and reproduction of many terrestrial taxa (Kronfeld‐Schor et al., 2013). Predator avoidance is typically observed in prey species around full moon (Griffin et al., 2005; Harmsen, Foster, Silver, Ostro, & Doncaster, 2011; Navarro‐Castilla & Barja, 2014; Palmer et al., 2017; Smith, Tulp, Schekkerman, Gilchrist, & Forbes, 2012), whereas other taxa exploit better foraging conditions during moonlit nights (Phalan et al., 2007, Mackley et al., 2011, Pinet et al., 2011, Penteriani et al., 2014, Rubolini et al., 2014, Dias et al., 2016, Roeleke et al., 2018; but see Cruz et al., 2013). Our study shows that the nocturnal flight activity of European nightjars correlates with both the altitude of the moon and the fraction of illuminated moon. In line with previous findings (Mills, 1986), this suggests that low ambient light levels, that is, less than the light intensity of a quarter moon (0.01–0.03 lux (Kyba, Mohar, & Posch, 2017)), limit movement and/or foraging opportunities. Flying in a local area (Brigham & Barclay, 1992, Zwart et al., 2014) or commuting between breeding and foraging sites might be safer during lighter conditions, because of a reduced risk of colliding with dark objects (Cresswelll & Alexander, 1992; Evens et al., 2018). Moonlight may also increase prey visibility and hence foraging success, which is probably why nightjars invest most energy in territorial display and reproduction during periods with the greatest moonlight levels (Jackson, 1985; Mills, 1986; Perrins & Crick, 1996). Our study shows that nightjars are usually inactive during moonless parts of the night. Similarly, low light levels during moonless nights affected thermoregulation in several species of nightjars, whereby the birds entered torpor following reduced foraging opportunities (Brigham et al., 1999; Doucette, Brigham, Pavey, & Geiser, 2012; Smit et al., 2011). However, moonlit nights do not necessarily imply high foraging activity. For example, Afrotropical nightjars reduced their nocturnal activity during full moon, presumably in response to high predation risk (Brigham et al., 1999; Jetz et al., 2003).

Foraging success may be higher during moonlit nights if prey activity is higher than during dark nights (Jetz et al., 2003). Nightjars typically perch at the edge of open fields while foraging (Evens, Beenaerts, et al., 2017). The likelihood to detect and hawk flying insects silhouetted against the sky (Camacho, Palacios, Sáez, Sánchez, & Potti, 2014; Evens et al., 2018; Jackson, 2003) should be higher when the sky is illuminated (Ashdown & McKechnie, 2008; Jetz et al., 2003; Mills, 1986). Alternatively, prey might be harder to catch in moonlight, either because insects can better detect the predator (Penteriani, Kuparinen, del Delgado, & M., Lourenço, R. and Campioni, L., 2011) and make evasive manoeuvres or because insects fly higher and therefore nightjars would have to work harder to achieve the same net energy intake. Although the emergence of insects peaks around sunset and sunrise (Malmqvist et al., 2018), it has also been suggested that nocturnal insect activity is associated with near full moon (Jetz et al., 2003; Nowinszky, Petrányi, & Puskás, 2010). One study suggested that the nightly flight activity of Lepidopterans—the nightjars’ main food source—decreases during full moon nights (Raimondo, Strazanac, & Butler, 2004), whereas another study showed that the activity of species associated with open habitats increased during moonlit nights (Nowinszky, Kiss, Szentkirályi, Puskás, & Ladányi, 2012).

If nightjars are sensitive to relatively subtle changes in ambient light conditions, as our study suggests, we predict that artificial night lighting, especially skyglow during overcast nights (Jechow et al., 2017), will influence their behavior. Artificial night light can be perceived far from its source, even in uninhabited areas (Falchi et al., 2016), and is known to alter the behavior of many taxa, including insects (Altermatt & Ebert, 2016), reptiles (Brei et al., 2016), and birds (Cabrera‐cruz, Smolinsky, & Buler, 2018; Van Doren et al., 2017; Kempenaers, Borgström, Loës, Schlicht, & Valcu, 2010; Raap, Pinxten, & Eens, 2015; Silva, Samplonius, Schlicht, Valcu, & Kempenaers, 2014; Da Silva et al., 2015). Thus, in contrast to earlier suggestions (Sierro & Erhardt, 2019), artificial light at night potentially mimics conditions during moonlit nights, thereby potentially improving foraging conditions. Further studies can be designed to test whether and how environmental variation (e.g., cloud cover and temperature) and artificial light at night influence patterns of food availability (van Langevelde, Ettema, Donners, WallisDeVries, & Groenendijk, 2011) and individual behavior (Dominoni et al., 2013, Da Silva et al., 2015), and how this in turn affects population dynamics.

## CONFLICT OF INTERESTS

We declare we have no competing interests.

## AUTHOR CONTRIBUTION

**Ruben Evens:** Conceptualization (equal); Formal analysis (equal); Funding acquisition (equal); Investigation (lead); Methodology (equal); Visualization (equal); Writing‐original draft (lead). **Céline Kowalczyk:** Data curation (equal); Funding acquisition (equal); Resources (equal). **Gabriel Norevik:** Data curation (supporting); Resources (supporting); Validation (supporting). **Eddy Ulenaers:** Data curation (equal); Funding acquisition (equal); Investigation (equal). **Davaasuren Batmunkh:** Investigation (supporting). **Soddelgerekh Sodoo Bayargur:** Investigation (supporting). **Tom Artois:** Funding acquisition (equal); Investigation (supporting); Resources (supporting); Writing‐original draft (supporting). **Susanne Akesson:** Resources (supporting); Writing‐original draft (supporting). **Anders Hedenstrom:** Funding acquisition (supporting); Resources (supporting); Writing‐original draft (supporting). **Mihai Valcu:** Conceptualization (equal); Data curation (lead); Formal analysis (lead); Methodology (lead); Writing‐original draft (equal). **Bart Kempenaers:** Conceptualization (equal); Formal analysis (equal); Funding acquisition (equal); Methodology (equal); Resources (equal); Validation (lead); Writing‐original draft (equal).

## AUTHORS’ CONTRIBUTIONS

R.E and B.K.: Study conception and writing of initial draft. R.E and C.K.: Data collection. R.E.: Data analysis with the help of M.V.. All authors contributed to the methodology and subsequent versions.

## Supporting information

Appendix S1

## Data Availability

Data are available from the OSF Repository at: https://osf.io/cghx5/
