# Supplementary material for: Lunar synchronization of daily activity patterns in a crepuscular avian insectivore
Source: Ecol Evol. 2020 Jun 9;10(14):7106–16. doi: 10.1002/ece3.6412 (PMC7391349; doi:10.1002/ece3.6412)
Supplement: Supplementary file 1 — Appendix S1 [file ECE3-10-7106-s001.docx]

**Supplementary Materials**

*Supplementary Materials T1: Overview of deployment periods for recovered loggers.*

| **ID** | **Country** | **Tag type** | **Start** | **Stop** |
| --- | --- | --- | --- | --- |
| 22KT | Belgium | SOI-GDL3pam | 20/08/2018 | 20/05/2019 |
| 22BT | Mongolia | SOI-GDL3pam | 13/07/2018 | 01/04/2019 |
| 22BS | Mongolia | SOI-GDL3pam | 13/07/2018 | 05/03/2019 |
| X500 | Sweden | MDL | 15/07/2016 | 27/04/2017 |
| X506 | Sweden | MDL | 15/07/2016 | 23/05/2017 |
| X526 | Sweden | MDL | 15/07/2016 | 19/05/2017 |
| X572 | Sweden | MDL | 15/07/2016 | 25/05/2017 |
| X627 | Sweden | MDL | 15/07/2016 | 18/06/2017 |
| X630 | Sweden | MDL | 15/07/2016 | 30/05/2017 |
| XD86 | Sweden | MDL | 01/08/2017 | 08/06/2018 |
| XD87 | Sweden | MDL | 10/08/2017 | 10/06/2018 |

*Supplementary Materials 2: Actograms of all tracked individuals are available* [*from the OSF-depository: Actograms.html*](https://osf.io/cnzg6/)*. Actograms showing daily activity (white = inactive, colour = activity, height of coloured bar = activity level, i.e. measured activity per 60-min period). Each horizontal bar shows one day with time on the X-axis. Time is plotted in three-hour intervals and centred around midnight.*

*Supplementary Materials T2: Results of generalized mixed-effect models showing effects of time and date on nocturnal activity of 11 European nightjars from Belgium, Mongolia and Sweden at their breeding and non-breeding sites. See Methods for model details.*

| **Nocturnal activity** | | | | |
| --- | --- | --- | --- | --- |
| *Conditional model* | | | | |
| **Predictors** | **Estimate** | **SE** | **z** | **P** |
| Intercept | 0.048 | 0.377 | 0.13 | 0.9 |
| Time^a^ | -0.147 | 0.015 | -10.02 | < 0.0001 |
| Period^b^ | -0.790 | 0.120 | -6.61 | < 0.0001 |
| Date^c^ | 0.219 | 0.058 | 3.77 | 0.0002 |
| Previous activity^d^ | 0.027 | 0.004 | 7.63 | < 0.0001 |
| **Random effect** | **Variance** | **SD** | **Corr** |  |
| Individual ID (random intercept) | 2.583 | 1.607 |  |  |
| Moon within ID^e^ (random slope) | 6.361 | 2.522 | -0.97 |  |
| *Zero-inflation model* | | | | |
| **Predictors** | **Estimate** | **SE** | **z** | **P** |
| Intercept | -2.549 | 0.882 | -2.89 | 0.004 |
| **Random effect** | **Variance** | **SD** | **Corr** |  |
| Individual ID | 1.898 | 1.607 |  |  |

^a^ Standardized time per night.

^b^ Estimates for non-breeding compared to breeding.

^c^ Standardized date per season (breeding or non-breeding)

^d^ Activity during the previous 60-min period (to control for temporal autocorrelation).

^e^ Fraction of illuminated, visible moon per individual

.
